# Acceptance and commitment therapy for autistic adults: A randomized controlled pilot study in a psychiatric outpatient setting

**DOI:** 10.1177/13623613221140749

**Published:** 2022-12-13

**Authors:** Johan Pahnke, Markus Jansson-Fröjmark, Gerhard Andersson, Johan Bjureberg, Jussi Jokinen, Benjamin Bohman, Tobias Lundgren

**Affiliations:** 1 Karolinska Institutet and Region Stockholm, Sweden; 2 Linköping University, Sweden; 3 Stanford University, USA; 4 Umeå University, Sweden

**Keywords:** acceptance and commitment therapy, anxiety, autism, cognitive defusion, depression, interventions—psychosocial/behavioral, mindfulness, psychological flexibility, quality of life, stress

## Abstract

Autistic adults are at risk of stress-related psychiatric disorders and reduced life quality due to social, cognitive, and perceptual challenges. Mental health interventions adapted to autistic adults are scarce. Acceptance and commitment therapy has preliminarily indicated health benefits in autistic adults, although it has not been robustly evaluated. Overall, 39 adults (21 males; 21–72 years) with autism spectrum disorder and normal intellectual ability (*IQ M* = 108.5; *SD* = 13.5) were randomized to 14 weeks of adapted acceptance and commitment therapy group treatment (NeuroACT) or treatment as usual. The intervention was feasible. Perceived stress and quality of life (primary outcomes), alongside psychological inflexibility, cognitive fusion, cognitive and behavioral avoidance, and autistic mannerism were statistically significantly improved in NeuroACT compared with treatment as usual (*d* = 0.70–0.90). Clinically significant changes in perceived stress and quality of life were in favor of NeuroACT. Between-group altered depression, anxiety, sleep problems, one quality of life measure, functional impairment, social aspects of autism, and executive difficulties were statistically non-significant. Dropout was slightly higher in NeuroACT. NeuroACT may be a promising treatment for autistic adults with co-existing stress and reduced quality of life. More extensive studies are warranted to evaluate NeuroACT further.

**Lay abstract:**

Autistic adults are often stressed and feel depressed or anxious. However, mental health programs that are suited for autistic adults are few. Acceptance and commitment therapy is a psychotherapy method that seems to help people feel better, although not thoroughly evaluated in autistic individuals. In this study, 20 autistic adults had 14 weeks of acceptance and commitment therapy group treatment suited for autism (NeuroACT), while 19 autistic adults had ordinary care. The acceptance and commitment therapy group treatment program seemed logical and reasonable to the participants. Also, when comparing the participants in the NeuroACT group with those in the ordinary care group, the NeuroACT participants reported less stress and higher quality of life. Compared to the ordinary care group, they could also manage distressing thoughts better, perceived themselves as more flexible, and did not avoid stressful situations as much as before. However, there was no significant difference between the groups in depression, anxiety, sleep problems, social aspects of autism, everyday functioning, or executive challenges. Slightly more NeuroACT participants did not finish the treatment than ordinary care participants. In conclusion, the NeuroACT program may be a treatment for autistic adults who feel stressed and have reduced quality of life. More studies are needed to see how helpful the NeuroACT program is for autistic adults.

## Introduction

Autism spectrum disorder (ASD) is a neurodevelopmental condition characterized by challenges in social interaction, repetitive and restricted behavior and interest, and sensory hyper- and hyposensitivity (American Psychiatric Association (APA), 2013; Lai & Baron-Cohen, 2015; Lord et al., 2018). ASD is present in 1%–2% of the adult population (Idring et al., 2015). Executive difficulties (e.g. working memory, inhibition, or planning) often impair the ability to cope with daily hassles and reach long-term goals (Bednarz et al., 2020; Uddin, 2021; Wallace et al., 2016) and affect essential life areas, such as social relationships, work, and independent living (Bishop-Fitzpatrick et al., 2017; Brugha et al., 2016; Lai et al., 2020). Moreover, autistic adults have higher rates of perceived stress (Bishop-Fitzpatrick et al., 2017) and reduced quality of life (Park et al., 2019), alongside psychiatric symptoms (e.g. depression and anxiety) (Croen et al., 2015), problems with sleep (Morgan et al., 2020), and even premature mortality (Smith DaWalt et al., 2019). As many as 70% of autistic adults experience at least one lifetime depressive episode and 50% meet the criteria for a lifetime anxiety disorder (Lugnegård et al., 2011; Nah et al., 2018). Accordingly, the continuous development of feasible and effective treatments that address stress, quality of life, and psychological distress in autistic adults is paramount.

Many autistic individuals cannot tolerate or have limited effects from pharmacological treatments intended to impact psychiatric symptoms (LeClerc & Easley, 2015; Williams et al., 2013). Moreover, research on feasible and effective psychological interventions that address health outcomes in autistic adults is limited (Benevides et al., 2020). Common psychological treatments adapted for ASD that address mental health problems are cognitive behavior therapy (CBT) (Spain et al., 2015) and mindfulness-based stress reduction (MBSR) (Cachia et al., 2016). Mindfulness is an emotion regulation technique defined as non-judgmental and non-reactive attention to momentarily experiences, including thoughts, emotions, and body sensations (Dryden & Still, 2006; Guendelman et al., 2017; Ludwig & Kabat-Zinn, 2008). The results are promising; CBT has indicated improved anxiety, depression, and quality of life, and group-delivered interventions seem to be well-suited, supportive, and cost-efficient for an ASD population (Hesselmark et al., 2014; Spain et al., 2015; Weiss & Lunsky, 2010). MBSR adapted for ASD has shown health benefits in a range of areas, such as depression, anxiety, rumination, sleeping problems, and interpersonal sensitivity, and the effects seem to last at least 9 weeks after treatment completion (Kiep et al., 2015; Spek et al., 2013). Furthermore, Conner and White (2018) have observed increased emotion regulation and impulse control in autistic adults as a result of mindfulness practice. Psychological treatments based on CBT and mindfulness principles thus appear to have the potential to benefit mental health in autistic adults. Also, given the heterogeneity across the autism spectrum, such as cognitive profile, functional level, or comorbidity, new treatment models are continuously warranted to cover different aspects of autism, increase mental health, and optimize everyday life in autistic individuals.

Acceptance and commitment therapy (ACT) is a psychotherapy method within the CBT umbrella that combines mindfulness procedures and behavioral change techniques (Villatte et al., 2016). ACT has been proven to reduce psychological distress in complex and persistent conditions, such as chronic pain, epilepsy, and psychosis (Hayes, 2019; Hughes et al., 2017; Shawyer et al., 2017). ACT may complement existing psychological treatments for mental health problems in autism by targeting purported underlying mechanisms somewhat differently than CBT and MBSR. In ACT, psychological inflexibility means that an individual avoids situations that are perceived as stressful or unpleasant, including thoughts, emotions, and body sensations related to those situations (Hayes & Wilson, 1994). Although sometimes helpful in the short run, repeated avoidance often implies missing out on essential things in life, eventually leading to a sense of hopelessness and causing or worsening mental health problems. In addition, autistic individuals are more exposed to stressors, such as aversive sensory experiences and stressful social situations. Therefore, they tend to run a higher risk of developing avoidant behaviors and maladaptive coping strategies, which might cause long-term mental health problems (Conner et al., 2020; Pagni et al., 2020; Pfaff & Barbas, 2019). ACT’s goal for autistic individuals is not to “treat autism” but to facilitate everyday life regardless of autistic core challenges. Also, research indicates that autistic core difficulties, such as social motivation, are not independent of emotional distress, suggesting that reducing stress in autistic individuals may affect how the individual perceives his or her difficulties (Hirvikoski & Blomqvist, 2015; South et al., 2017). Therefore, increasing coping skills to handle stressful situations more flexibly could reduce avoidance and benefit mental health and quality of life in autistic adults.

In ACT, psychological flexibility is the ability to do what is essential to oneself while handling mental obstacles that would otherwise be in the way (Hayes et al., 2006). Psychological flexibility is enhanced mainly through two procedures: (1) training mindfulness, cognitive defusion, and acceptance skills, and (2) using behavior change techniques. Cognitive defusion is the ability to observe thoughts without literally believing their content or letting them guide one’s actions (Gillanders et al., 2013). Acceptance is the active and aware embracement of thoughts, emotions, and body sensations without attempts to avoid or counteract them, especially when doing so would cause psychological harm (Hayes et al., 2011). Mindfulness, cognitive defusion, and acceptance help the individual to cope with stressful thoughts (e.g. “I’m worthless”), emotions (e.g. fear or sadness), and body sensations (e.g. heart palpitation), thereby preventing avoidance. Behavioral change techniques assist the individual in defining values, which is what is important to him or her (e.g. social contact), and acting according to this (e.g. texting a friend or using public transport), thereby reaching personally chosen behavior goals.

Interventions based on ACT have been evaluated for parents of autistic children (Hahs et al., 2019; Prevedini et al., 2020; Whittingham et al., 2020), autistic adolescents (Pahnke et al., 2014), and autistic adults in a non-clinical setting (Hutchinson et al., 2019). However, only a few studies have evaluated ACT-based interventions for autistic adults in a clinical context. For example, Pahnke et al. (2019) found preliminary benefits of an ACT protocol adapted for autistic adults on perceived stress, quality of life, and depression, alongside reduced psychological inflexibility and cognitive fusion. Moreover, Maisel et al. (2019) showed reduced psychological distress in autistic adults using a cognitive defusion intervention. However, to our knowledge, there is no randomized controlled trial (RCT) of ACT for autistic adults. Therefore, studies using an RCT design to evaluate the feasibility and effectiveness of ACT adapted to autistic adults are of importance.

### Study objectives

The current pilot RCT study evaluated the feasibility and preliminary effectiveness of an ACT group protocol (NeuroACT) adapted for autistic adults in a psychiatric outpatient setting compared with treatment as usual (TAU). The research questions were as follows: (1) Is the study procedure and the NeuroACT protocol feasible (i.e. treatment completion, treatment credibility, data collection, and participant recruitment)? (2) What are the effects of NeuroACT on perceived stress and quality of life (primary outcomes) compared with TAU? (3) What are the effects of NeuroACT on psychiatric symptoms (i.e. depression, anxiety, and sleep problems) and functional impairment (secondary outcomes) compared with TAU? (4) What are the effects of NeuroACT on ACT-related variables (i.e. psychological inflexibility, cognitive fusion, and cognitive and behavioral avoidance (secondary outcomes)) compared with TAU? (5) How does NeuroACT affect the individual’s perception of his or her autistic core challenges, such as social motivation, social awareness, social cognition, communication, autistic mannerism (i.e. cognitive and behavioral inflexibility), and executive difficulties (secondary outcomes), compared with TAU?

## Methods

### Design

The study design was a randomized two-group controlled pilot trial with repeated measures evaluating the feasibility and preliminary effectiveness of NeuroACT compared with TAU for autistic adults. The pilot study design allowed for the inclusion of several outcome measures to cover different aspects of potential treatment benefits and to follow a broad spectrum of symptoms, correlates, and consequences of autism. Assessments were conducted at pre-treatment (T1), post-treatment (T2), and 6 months after treatment completion (T3). Power calculation was based on an open-trial pilot study on ACT for autistic adults (Pahnke et al., 2019) using the results of the Satisfaction with Life Scale (SWLS) (Diener et al., 1985) (*M* = 13.2, *SD* = 5.1 for pre-treatment and *M* = 17.0, *SD* = 4.8 for post-treatment, 0.05 significance level, and 0.8 statistical power). The calculation indicated the need for 30 participants in each treatment group (a total of 60 participants) to reduce the risk of Type 2-error. However, due to organizational changes within the psychiatric clinic, the study was prematurely aborted, reaching a total of 39 participants.

**Figure 1.**
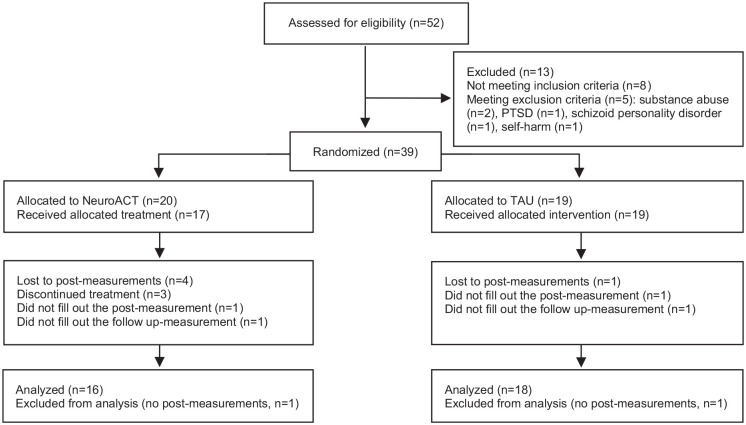
Flowchart of procedure and participants.

Randomization was conducted block-wise (Schochet et al., 2021) and performed 1:1 using folded pieces of paper reading either “treatment” or “control” placed in a container, mixed, and drawn. Overall, 20 participants were randomized in the first block (fall 2011) and 19 were randomized in the second block (fall 2012). Post-assessment (T2) was conducted 1 week after treatment completion, and a follow-up assessment (T3) was performed 6 months after the post-assessment. As shown in Figure 1, 52 adults were screened for the study, 13 were not found eligible, and 39 (75%) were included.

### Ethics

The trial was approved by the regional ethics committee of Stockholm, Sweden (2015-1005-31) and followed the Declaration of Helsinki (American Medical Association (AMA), 2013). All participants were given verbal and written information about the study procedures and that they could withdraw from study participation at any time without further explanation.

### Participants

Participants were recruited at the Neuropsychiatric Unit Karolinska, Psychiatry Northwest, Stockholm City Council, Sweden, a clinic specialized in assessing and treating neurodevelopmental disorders in adults. Individuals who met the diagnostic criteria of ASD were invited to participate. Inclusion criteria were as follows: (a) a diagnosis of *Diagnostic and Statistical Manual of Mental Disorders* (4th ed.; DSM-IV) Asperger’s syndrome (i.e. equivalent to ASD without specified intellectual disability or language impairment in the *Diagnostic and Statistical Manual of Mental Disorders* (5th ed.; DSM-5; APA, 2013) as the primary neurodevelopmental diagnosis; (b) 18 years of age or older; (c) if on any psychoactive drug treatment (for attention deficit hyperactivity disorder (ADHD) or other diagnoses), treatment should have been stable (at least for 3 months); and (d) scoring more than one standard deviation under the population mean on the Quality of Life Inventory (QOLI) (Frisch, 1994), that is, QOLI < 1.84 or more than one standard deviation over the population mean on the Perceived Stress Scale (PSS) (S. Cohen et al., 1983), that is, PSS > 24. Exclusion criteria were as follows: (a) ongoing substance abuse (last 3 months); (b) diagnosed intellectual disability (intelligence quotient, IQ < 70); (c) organic brain injury; (d) suicidality; and (e) severe clinically unstable psychosocial circumstances or comorbid psychiatric disorders (e.g. being homeless or severe depression, psychosis, or bipolar disorder not under stable pharmacological treatment). An explicit study objective was to include a representative selection of psychiatric patients with ASD. Hence, comorbid neurodevelopmental disorders (e.g. ADHD or Tourette’s disorder) were not excluded. Participants included were 21 men and 18 women (21–72 years) with a mean age of 39 years (*SD* = 12). The ACT group consisted of 20 participants (10 males), and the TAU group contained 19 participants (11 males).

### Assessment

The diagnostic assessment followed local clinical guidelines and was based on multiple sources of information. First, a clinical interview was performed by a psychiatrist, followed by neuropsychological testing by a psychologist (e.g. Wechsler Adult Intelligence Scale–Revised (WAIS-R), or Wechsler Adult Intelligence Scale–Third Edition (WAIS-III)) (Wechsler, 1981, 1997), frequently complemented by Conners’ Continuous Performance Test (CPT-II) (Conners, 2000) or Delis–Kaplan Executive Function System (D-KEFS) (Delis et al., 2001). Second, assessing autistic and ADHD symptoms included standardized self-rating questionnaires (e.g. Adult Autism Spectrum Quotient (AQ); Wender Utah Rating Scale (WURS); Baron-Cohen et al., 2001; Ward et al., 1993). Third, family members or significant others were interviewed for a complete medical history, and information was obtained from child and adolescent psychiatry, school health services, and adult psychiatry. Fourth, demographic and clinical data were obtained from medical records and a self-report questionnaire covering different clinical aspects (Hirvikoski et al., 2009). Finally, feasibility and outcome self-report questionnaires were administered by clinical psychologists to evaluate the treatment.

### Treatment

The manualized treatment (NeuroACT—stress management for flexibility and health) was a modified version of the protocol evaluated in autistic adults within a psychiatric outpatient setting (Pahnke et al., 2019). The neuroACT treatment manual can be accessed from the website http://www.brainproof.se or by contacting the corresponding author. The treatment program consists of training in ACT processes, psychoeducation on stress, emotions, and perception, and support for executive difficulties. Primary treatment objectives were to (1) facilitate participants’ motivation to behavior change and (2) train participants’ skills to cope with daily hassles and stressful situations to reduce behavioral avoidance. The treatment consisted of 14 weekly 150-min group sessions with 8–10 participants, led by two clinical psychologists with experience in ACT and autism (the first and fourth authors of this article). After each session, 30 min were added for questions or assistance with homework assignments. Each session had a similar format with a short mindfulness or acceptance exercise, followed by a review of homework assignments, an introduction to the theme of the particular session, and a review of new homework assignments and session evaluation. In-session activities and homework assignments consisted of pencil-and-paper exercises using adapted worksheets (i.e. recording stressful situations and avoidance behaviors, values and actions work, cognitive defusion exercises, and visualized metaphors). In addition, mindfulness and acceptance were practiced at home five times per week using prerecorded adapted audio exercises. Before each exercise, a rationale for why to practice mindfulness or acceptance was provided. The central components and processes of each treatment session were explained using didactic presentations. In addition, psychoeducational information sheets were provided, such as about stress, emotions, or perception. Compared to the protocol used by Pahnke et al. (2019), modifications consisted of two additional sessions to enhance problem-solving and everyday-structure skills. Central treatment components and aims are described in Table 1.

**Table 1.** NeuroACT treatment modules and sessions.

**Module 1. Stress and avoidance (Session 1–2)** Psychoeducation on stress from an ACT perspective. • Recording of stressful situations. Avoidance trap.	**Module 2. Perspective-taking (Session 3–4)** • Introduction to mindfulness and cognitive defusion. • Being present. Perspective-taking skills.
**Module 3. Values and committed action (Session 5–6)** • Values and motivation work. • Purpose and meaning. Behavior goals and committed action.	**Module 4. Acceptance and compassion (Session 7–8)** • Acceptance and compassion skills. • Acceptance of emotions and body sensations. Acceptance of sensory input.
**Module 5. Integration of ACT (Session 9–10)** • Using presence, defusion, and acceptance. • Managing stress in social situations. • Restorative actions. Module 7. Consolidation of ACT (Session 13–14) • Action plan. • Review of group experiences. Planning for the future.	**Module 6. Support of executive function (Session 11–12)** • Problem-solving. • Structure management. Application of ACT techniques.

ACT: acceptance and commitment therapy.

The TAU group received ordinary care, such as communication training, psychoeducational programs, or psychotherapy, as part of their standard disability service or outpatient psychiatric care and obtained the NeuroACT treatment with a 1-year delay.

### Measures

#### Assessment measures

##### Intellectual ability

Intellectual ability (IQ) was assessed using the WAIS-R (Wechsler, 1981) or the WAIS-III (Wechsler, 1997). WAIS consists of verbal and performance subtests where a verbal IQ, a performance IQ, and a full-scale IQ are obtained. The population mean of IQ and index scores is 100, with a standard deviation of 15. WAIS’ test–retest reliability ranges between 0.70 and 0.90, inter-scorer coefficients are high (*r* = 0.90), and WAIS’ full-scale IQ correlates highly with the Stanford–Binet IV test (*r* = 0.88) (Wechsler, 1981).

##### Psychiatric diagnoses

Comorbid psychiatric disorders were assessed using the Mini-International Neuropsychiatric Interview (MINI) (Sheehan et al., 1998), a structured diagnostic interview for DSM and *International Classification of Disease* (ICD) psychiatric disorders. MINI has shown moderate concurrent validity with mood and anxiety disorders (Verhoeven et al., 2017) with AUC (i.e. area under the receiver operating characteristic curve) ranging between 0.55 and 0.81 (median 0.73) for mood disorders and between 0.78 and 0.88 (median 0.83) for anxiety disorders (Verhoeven et al., 2017).

##### Feasibility measures

Overall feasibility was calculated as a percentage of (1) treatment completion; (2) measurement fulfillment at T1, T2, and T3; (3) dropout rates between the NeuroACT group and the TAU group post randomization; (4) treatment credibility; and (5) any adverse events as reported in participants’ medical records, according to the CONSORT statement for randomized trials of nonpharmacological treatments (Boutron et al., 2017).

Treatment credibility was assessed using an ASD-adapted version of the Treatment Credibility Scale (TCS) (Borkovec & Nau, 1972). TCS consists of five items scored on a scale from 1 to 10, with a higher score indicating more credibility of the current treatment. In addition, items were adjusted to be relevant for autistic individuals: (1) how apprehensible the treatment seemed to the participants; (2) how confident they felt that the group would reduce their ASD-related problems; (3) how confident they would be in recommending this kind of group to a friend with ASD; (4) how successful the participants thought that the treatment would be for other diagnoses, and (5) how much improvement they expected to become with this treatment. The TCS total score is calculated as a mean of all items. The TCS has demonstrated good internal consistency in a Swedish sample consisting of stress and anxiety patients (Cronbach’s α = 0.83) (Alfonsson et al., 2016) and satisfactory internal consistency in the current sample (Cronbach’s α = 0.92).

#### Primary outcome measures

##### Stress

Perceived stress was assessed using the PSS 14 items (PSS-14) (Cohen et al., 1983), a 14-item five-point Likert-type scale (0 = *never* to 4 = *very often*), with higher scores indicating more stress. A total score is calculated after reversing positive items’ scores and then summing up all scores. The PSS has shown good criterion validity with anxiety (*r* = 0.68), depression (*r* = 0.57), and mental or physical exhaustion (*r* = 0.71), and good internal consistency in a Swedish sample (Cronbach’s α = 0.84) (Eklund et al., 2014; Nordin & Nordin, 2013). The PSS showed satisfactory internal consistency in the present sample (Cronbach’s α = 0.77).

##### Quality of life

Perceived quality of life was assessed using the SWLS (Diener et al., 1985) and the QOLI (Frisch et al., 1992). The SWLS consists of five items rated on a Likert-type scale 1–7, with a higher score indicating higher quality of life. A total score is calculated as the sum of the item scores. Satisfactory convergent validity (*r* = 0.39) (Glaesmer et al., 2011) and good internal consistency (Cronbach’s α = 0.88) of the SWLS in a Swedish clinical sample have been observed (Hultell & Gustavsson, 2008). The QOLI assesses 16 life areas, presenting a weighted score considering each domain’s importance and satisfaction. The QOLI has shown satisfactory to good internal consistency (Cronbach’s α = 0.77–0.89) and test–retest reliability (Frisch et al., 1992). In addition, the SWLS showed satisfactory internal consistency (Cronbach’s α = 0.74) and the QOLI demonstrated good internal consistency in the current sample (Cronbach’s α = 0.88).

#### Secondary outcome measures

##### Depression

Perceived depressive symptoms were assessed using the Beck Depression Inventory–II (BDI-II) (A. T. Beck et al., 1961), a 21-item self-report four-point Likert-type scale, with higher scores indicating more depressive symptoms. A total score is calculated as the sum of the scores on each item. Good convergent validity (*r* = 0.72) (Lahlou-Laforêt et al., 2015) and internal consistency (Cronbach’s α = 0.89) were observed in a Swedish clinical sample (Kjærgaard et al., 2014). The BDI showed satisfactory internal consistency in the current sample (Cronbach’s α = 0.93).

##### Anxiety

Perceived anxiety symptoms were assessed using the Beck Anxiety Inventory (BAI) (A. T. Beck et al., 1988), a 21-item self-report four-point Likert-type scale, with a higher score indicating more anxiety symptoms. A total score is calculated as the sum of the scores on each item. Satisfactory internal consistency (Cronbach’s α = 0.91) and good test–retest-reliability (*r* = 0.84) have been reported (Vázquez-Morejón et al., 2014). The BAI showed satisfactory internal consistency in the current sample (Cronbach’s α = 0.95).

##### Sleep problems

Perceived sleep problems were assessed using the Karolinska Sleep Questionnaire (KSQ) (Kecklund & Åkerstedt, 1992), a 6-point Likert-type scale, with higher scores indicating more difficulties. KSQ covers four indices (i.e. sleep quality, awakening difficulties, breathing problems, and fatigue during daytime), which are recommended instead of the scale’s total score (Nordin & Nordin, 2013). The instrument has shown good criterion validity, internal consistency, and satisfactory construct validity in Swedish samples (Nordin & Nordin, 2013; Westerlund et al., 2014). In addition, the KSQ demonstrated satisfactory internal consistency in the present sample (Cronbach’s α = 0.91).

##### Functional impairment

Perceived functional impairment (familial, social, and vocational) was assessed using the Sheehan Disability Scale (SDS) (Sheehan et al., 1996), a three-item scale ranging from 0 to 10, with a higher score indicating more functional impairment. The SDS has shown satisfactory AUC statistics (0.81) (Luciano et al., 2010) and good internal consistency (Cronbach’s α = 0.89) (Leon et al., 1997). In addition, good internal consistency of the SDS was observed in the present sample (Cronbach’s α = 0.79).

##### Psychological inflexibility

Perceived psychological inflexibility was assessed using the Acceptance and Action Questionnaire (AAQ-7) (Bond et al., 2011), a seven-item Likert-type scale (1–7), with a higher score indicating more psychological inflexibility. AAQ was evaluated in a Swedish sample showing good concurrent and convergent validity, and good internal consistency (Cronbach’s α = 0.85) and test–retest reliability (*r* = 0.80) (Lundgren & Parling, 2017). In addition, the AAQ showed satisfactory internal consistency in the present sample (Cronbach’s α = 0.92).

##### Cognitive fusion

Perceived cognitive fusion was assessed using the Cognitive Fusion Questionnaire (CFQ-7) (Gillanders et al., 2013), a 7-item Likert-type scale (1–7), with a higher score reflecting more cognitive fusion. Discriminative validity of the CFQ against psychological acceptance has been observed as satisfactory (*r* = −0.78) in a clinical sample (McCracken et al., 2014). In addition, the CFQ demonstrated satisfactory internal consistency in a clinical sample (Cronbach’s α = 0.93) (Ruiz et al., 2017) and in the current sample (Cronbach’s α = 0.93).

##### Cognitive and behavioral avoidance

Perceived cognitive and behavioral avoidance was assessed using the Cognitive–Behavioral Avoidance Scale (CBAS) (Ottenbreit & Dobson, 2004), a 31-item 5-point Likert-type scale, with a higher score indicating more cognitive and behavioral avoidance. The CBAS has demonstrated satisfactory internal consistency (Cronbach’s α = 0.95), good convergent and discriminative validity, and good 4-month test–retest reliability in psychiatric samples (Barajas et al., 2017; Ottenbreit & Dobson, 2004). In the present sample, the CBAS showed good internal consistency (Cronbach’s α = 0.89).

##### Autistic core challenges

To assess the participants’ subjective perception of their autistic core challenges, we used the Social Responsiveness Scale (SRS) (Constantino, 2002), a 65-item 4-point Likert-type scale resulting in a total score and five subscale scores (social motivation, social cognition, social awareness, social communication, and autistic mannerism (AM)). Social motivation assesses the degree of motivation to participate in social-interpersonal behavior. Social cognition measures the ability to understand social information, while social awareness implies noticing social cues. Social communication refers to the ability to demonstrate expressive social communication. Finally, AM refers to cognitive and behavioral inflexibility (Booker & Starling, 2011). The SRS showed good concurrent, predictive, convergent, and discriminative validity with a sensitivity of 0.85 and a specificity of 0.83 for ASD and a Cronbach’s alpha of 0.89 in different ASD populations (Bolte, 2012; Chan Smith et al., 2017; Frazier et al., 2014). In addition, the SRS showed satisfactory internal consistency in the current sample (Cronbach’s α = 0.91). In accordance with recommended research practices, we used raw scores for all analyses.

##### Executive difficulties

Perceived executive difficulties were assessed using the Dysexecutive Questionnaire (DEX-S), a 20-item 5-point Likert-type scale, with a higher score indicating more executive problems (Wilson et al., 1996). The DEX-S showed satisfactory internal consistency (Cronbach’s α = 0.91) for neurologically impaired patients (Bennett et al., 2005; Shaw et al., 2015) and good internal consistency in the current sample (Cronbach’s α = 0.84).

### Statistical analyses

Data were analyzed using the statistical software SPSS version 27.0. Demographic data and background variables were analyzed using independent *t-*tests for continuous variables and chi-square tests for categorical variables. An exploratory analysis was performed to assess the normal distribution and potential outliers, indicating normality for all measures except the BDI, the BAI, the KSQ—Breathing Index, and the KSQ—Fatigue Index. Outcome measures were analyzed using two-tailed mixed-design repeated-measures analyses of variance (rmANOVA). Group (NeuroACT/TAU) was the between-subjects factor and time (T1, T2, and T3) was the within-subjects factor. Calculations of the outcome measures were performed on treatment completers. Contrast analyses were performed from pre (T1)- to post (T2)-intervention and from post- to six-month follow-up (T3). Kruskal–Wallis and Wilcoxon Signed-rank tests were used for non-normal distributed samples. The interpretations of Cronbach’s alpha were α ⩾ 0.70 = *satisfactory*, α ⩾ 0.80 = *good*, and α ⩾ 0.90 = *satisfactory*, where a too low or high alpha value may indicate insufficient reliability (Taber, 2018). Effect sizes were calculated by converting R-squared effect size to Cohen’s *d*, interpreted using the guidelines proposed by J. Cohen (1988): 0.2 = *small effect size*, 0.5 = *moderate effect size*, and 0.8 = *large effect size*. Alpha levels were set at *p* ⩽ 0.05 for statistical significance.

Clinically significant changes (Evans et al., 1998; Jacobson & Truax, 1991) in the primary outcome measures were calculated using normal population data of the PSS (*M* = 24.8, *SD* = 11.1) (Eklund et al., 2014) and the SWLS (*M* = 24.1, *SD* = 6.9) (Pavot & Diener, 2008) along with clinical data from the present sample. In the PSS, a cut-off score below 31.36 for the NeuroACT group and below 30.35 for the TAU group was interpreted as a clinically significant recovery. A change score below two standard deviations (NeuroACT = 15.0; TAU = 15.4) of the group mean (NeuroACT = 35.8; TAU = 34.2) was interpreted as a clinically significant improvement, and within two standard deviations from the group mean was interpreted as unimproved. For the SWLS, a cut-off score above 18.5 for the NeuroACT group and above 18.4 for the TAU group was interpreted as a clinically significant recovery. A change score exceeding two standard deviations (NeuroACT = 10.2; TAU = 9.8) of the group mean (NeuroACT = 14.3; TAU = 14.4) was interpreted as a clinically significant improvement, and within two standard deviations from the group was interpreted as unimproved.

## Results

### Participant characteristics

A slight majority of participants were male (54%). Most had comorbid disorders (56%; e.g. ADHD/attention deficit disorder (ADD), depression, or dysthymia) and used pharmacological treatment (72%; e.g. antihistamines, sleep medication, antidepressants, or methylphenidate). The mean overall IQ score was 108.5 (*SD* = 13.5). The NeuroACT group had an IQ score of 107.0 (*SD* = 13.8), and the TAU group’s IQ score was 109.6 (*SD* = 13.9). The distribution of participant characteristics (e.g. age, sex, IQ, psychiatric comorbidity, medication, and occupation) is shown in Table 2.

**Table 2.** Participant characteristics.

Characteristics	NeuroACT (*n* = 20)		TAU (*n* = 19)		Total (*N* = 39)	
	*n*		*n*		*n*	
Gender, male	10		11		21	
Age (years)	*M* 38.4	(*SD*) (10.0)	*M* 39.8	(*SD*) (14.4)	*M* 39.1	(*SD*) (12.2)
Psychiatric comorbidity						
ADHD/ADD	9		6		15	
Depression, depressive episode NOS, dysthymia	4		5		9	
Anxiety disorders	5		0		5	
Other comorbidities (e.g. dyslexia; bipolarity)	5		3		8	
Any psychiatric comorbidity	11		11		22	
Medication						
Antihistamines	2		7		9	
Sleep medication	4		5		9	
Antidepressants	5		8		13	
Methylphenidate	5		7		12	
Other medication	6		11		17	
Any medication	13		16		29	
Education						
University/higher education	7		3		10	
High school	9		12		21	
Elementary school	3		3		6	
Other	1		1		2	
Occupation						
Company owner/employee/student/parental leave	7		5		12	
Part-time employee/temporary position	2		3		5	
Pensioner	0		1		1	
Unemployed	2		4		6	
Temporary disability pension/early retirement benefit	5		3		8	
Other	4		3		7	

ACT: acceptance and commitment therapy; TAU: treatment as usual; ADHD: attention deficit hyperactivity disorder; ADD: attention deficit disorder; NOS: not otherwise specified.

### Feasibility

Results showed good overall feasibility: 39 out of 52 assessed participants (75%) were considered candidates and invited to the study. All 39 invited participants chose to participate. However, 17 of 20 participants (85%) completed the treatment. Meanwhile, 34 of 39 (87%) completed all assessments at T1, T2, and T3. Dropout rates were slightly higher in the NeuroACT group (20%) compared with the TAU group (5%) post randomization, and no adverse events were reported. Two participants were included who had a lower score (20 and 23, respectively) on the PSS than the inclusion cut-off (PSS > 24) but instead met the inclusion criteria on QOLI. Five participants were included who had a higher score (1.9–2.9) in the QOLI than the inclusion cut-off (QOLI < 1.84) but instead met inclusion criteria on PSS.

Treatment credibility (max score = 10) was rated as high (*M* = 7.3, *SD* = 2.5) using the TCS (Borkovec & Nau, 1972). The mean score of the TCS was 7.6 (*SD* = 2.5) on Item 1 (how apprehensible the treatment seemed to the participants); 6.3 (*SD* = 3.2) on Item 2 (how confident they felt that the group would reduce their ASD-related problems; 7.9 (*SD* = 3.2) on Item 3 (how confident they would be in recommending this kind of group to a friend with ASD); 8.0 (*SD* = 2.7) on Item 4 (how successful the participants thought that the treatment would be for other diagnoses); and 6.4 (*SD* = 3.1) on Item 5 (how much improvement they expected to become with this treatment).

### Primary outcomes

#### Stress and quality of life

As presented in detail in Table 3, the results of the rmANOVA showed a statistically significant group-by-time interaction effect in favor of the NeuroACT group compared with the TAU group on perceived stress (PSS), with a moderate effect size. Contrast analyses showed a statistically significant reduction in perceived stress from T1 to T2 but not from T2 to T3 in the NeuroACT group compared with the TAU group. In addition, the results showed a statistically significant interaction effect, with moderate effect size, in favor of the NeuroACT group compared with TAU on one quality of life measure (SWLS) but not the second quality of life instrument (QOLI). Contrast analyses showed no statistically significant group-by-time change between the two groups in quality of life (SWLS) from T1 to T2 or T2 to T3.

**Table 3.** Means, standard deviations, statistical significance, and effect sizes between groups for stress, quality of life, sleep problems, and functional impairment at pre, post, and 6-month follow-up.

Measure	*n*	Pre	Post	6 months	Group-by-time interaction effect (within-subjects)		Pre–post	Post 6 months
	NeuroACT = 16 TAU = 18	*M* (*SD*)	*M* (*SD*)	*M* (*SD*)	ANOVA	*d*	*d*	*d*
PSS	NeuroACT	35.8 (7.5)	24.9 (8.4)	22.6 (8.1)	*F* (2, 64) = 4.60	0.76*	**1.02** **	0.40
	TAU	34.2 (7.7)	32.3 (8.6)	28.8 (8.1)				
SWLS	NeuroACT	14.3 (5.1)	18.7 (5.9)	20.3 (5.6)	*F* (2, 64) = 3.85	0.77*	0.69	0.71
	TAU	14.4 (4.9)	15.4 (6.2)	16.4 (5.7)				
QOLI	NeuroACT	0.70 (1.6)	1.67 (1.4)	1.41 (1.7)	*F* (2, 64) = 1.35	0.41	0.50	0.26
	TAU	−0.21 (1.5)	0.12 (1.9)	0.44 (1.7)				
KSQ-S	NeuroACT	10.8 (6.6)	7.7 (6.6)	6.9 (5.1)	*F* (2, 64) = 3.12	0.63	0.78	0.11
	TAU	9.2 (6.5)	9.8 (5.5)	7.5 (5.0)				
KSQ-A	NeuroACT	7.8 (4.4)	7.4 (4.5)	5.7 (5.1)	*F* (2, 64) = 0.57	0.26	0.19	0.37
	TAU	7.3 (4.6)	7.8 (4.5)	6.7 (3.8)				
SDS	NeuroACT	17.6 (6.1)	14.6 (4.9)	15.1 (7.3)	*F* (2, 64) = 1.47	0.43	0.59	0.09
	TAU	19.4 (6.5)	19.7 (5.4)	19.1 (5.9)				

ANOVA: analysis of variance; PSS: Perceived Stress Scale; SWLS: Satisfaction with Life Scale; QOLI: Quality of Life Inventory; BDI-II: Beck Depression Inventory–II; KSQ-S: Karolinska Sleep Questionnaire–Sleep quality Index; KSQ-A: Karolinska Sleep Questionnaire–Awakening Index; SDS: Sheehan Disability Scale. Effect size measured by Cohen’s *d* (0.2 = *small*; 0.5 = *moderate*; 0.8 = *large*).

* *p* < 0.05. **p < 0.01.

### Secondary outcomes

#### Psychiatric symptoms and functional impairment

As shown in Table 3, the results of the rmANOVA showed no statistically significant group-by-time interaction effect in sleep quality (KSQ-S), awakening problems (KSQ-A), or functional impairment (SDS) in the NeuroACT group compared with the TAU group. In addition, Kruskal–Wallis tests showed no group-by-time statistically significant interaction effect between the two groups in depressive symptoms (BDI), anxiety (BAI), breathing problems (KSQ-Breathing Index), or fatigue during daytime (KSQ-Fatigue Index).

#### Psychological inflexibility, cognitive fusion, and cognitive and behavioral avoidance

As shown in Table 4, statistically significant group-by-time interaction effects were found in measures of psychological inflexibility (AAQ), CFQ, and CBAS, with moderate to large effect sizes in the NeuroACT group compared with the TAU group. In addition, contrast analyses showed significant improvements to occur from T1 to T2 in all measures in the NeuroACT group compared with the TAU group.

**Table 4.** Means, standard deviations, statistical significance, and effect sizes between groups for ACT-related measures at pre, post, and 6-month follow-up.

Measure	*n*	Pre	Post	6 months	Group-by-time interaction effect (within-subjects)		Pre–post	Post 6 months
	NeuroACT = 16 TAU = 18	*M* (*SD*)	*M* (*SD*)	*M* (*SD*)	ANOVA	*d*	*d*	*d*
AAQ	NeuroACT	30.4 (11.0)	23.9 (9.4)	21.6 (9.1)	*F* (2, 64) = 3.91	0.70*	0.79*	0.61
	TAU	30.4 (9.5)	29.8 (9.9)	28.7 (8.5)				
CFQ	NeuroACT	33.5 (11.4)	25.0 (9.4)	24.3 (8.0)	*F* (2, 64) = 5.32	0.82**	1.07**	0.35
	TAU	31.0 (9.2)	31.6 (9.9)	28.7 (8.8)				
CBAS	NeuroACT	82.1 (21.1)	67.0 (19.6)	65.3 (22.1)	*F* (2, 64) = 6.44	0.90**	1.24**	0.41
	TAU	80.7 (15.4)	86.5 (18.0)	80.7 (16.0)				

ANOVA: analysis of variance; AAQ: Acceptance and Action Questionnaire–7 items; CFQ: Cognitive Fusion Questionnaire–7 items; CBAS: Cognitive–Behavioral Avoidance Scale. Effect size measured by Cohen’s *d* (0.2 = *small*; 0.5 = *moderate*; 0.8 = *large*).

* *p* < 0.05. **p < 0.01.

#### Autistic core challenges

As shown in Table 5, a statistically significant group-by-time interaction effect was observed in AM (SRS-AM), with a moderate effect size, in favor of the NeuroACT group compared with the TAU group. Contrast analyses showed a statistically significant reduction in AM from T2 to T3 but not from T1 to T2 in the NeuroACT group compared with the TAU group. No statistically significant group-by-time interaction effect was observed between the two groups in overall autistic core challenges (SRS total score), social motivation (SRS-M), social awareness (SRS-A), social cognition (SRS-SC), communication (SRS-C), or executive difficulties (DEX).

**Table 5.** Means, standard deviations, statistical significance, and effect sizes between groups for autistic core challenges and executive difficulties at pre, post, and 6-month follow-up.

Measure	*n*	Pre	Post	6 months	Group-by-time interaction effect (within-subjects)		Pre–post	Post 6 months
	NeuroACT = 16 TAU = 18	*M* (*SD*)	*M* (*SD*)	*M* (*SD*)	ANOVA	*d*	*d*	*d*
SRS	NeuroACT	89.5 (28.1)	80.4 (22.7)	70.0 (28.7)	*F*(2, 64) = 2.55	0.57	0.43	0.62
	TAU	88.6 (20.0)	86.1 (15.7)	83.5 (16.7)				
SRS-AM	NeuroACT	15.8 (7.4)	12.1 (5.7)	10.6 (6.0)	*F* (2, 64) = 3.93	0.70*	0.64	0.75*
	TAU	15.6 (6.2)	14.8 (6.0)	15.1 (5.2)				
SRS-M	NeuroACT	17.9 (6.0)	14.8 (4.7)	12.5 (6.6)	*F*(2, 64) = 2.92	0.61	0.61	0.60
	TAU	17.8 (5.4)	17.5 (4.3)	16.4 (3.8)				
SRS-A	NeuroACT	10.1 (3.9)	9.9 (3.2)	8.9 (3.0)	*F*(2, 64) = 0.14	0.13	0.01	0.16
	TAU	9.6 (3.0)	9.2 (3.0)	8.8 (3.0)				
SRS-SC	NeuroACT	16.6 (4.6)	16.5 (6.9)	14.7 (6.5)	*F*(2, 64) = 0.94	0.35	0.00	0.46
	TAU	15.9 (4.2)	15.7 (3.6)	15.7 (4.5)				
SRS-C	NeuroACT	29.1 (10.2)	27.1 (7.8)	23.3 (11.4)	*F*(2, 64) = 1.27	0.40	0.19	0.49
	TAU	29.7 (7.0)	28.8 (6.4)	27.6 (6.6)				
DEX	NeuroACT	37.8 (11.5)	31.6 (11.7)	28.2 (8.6)	*F*(2, 64) = 2.04	0.51	0.41	0.43
	TAU	37.3 (9.9)	36.8 (11.4)	34.1 (8.4)				

TAU: treatment as usual; ANOVA: analysis of variance; SRS: Social Responsiveness Scale–total score; SRS-AM: Social Responsiveness Scale–Autistic mannerism; SRS-M: Social Responsiveness Scale–Motivation; SRS-A: Social responsiveness Scale–Social Awareness; SRS-SC: Social Responsiveness Scale–Social Cognition; SRS-C: Social Responsiveness Scale–Communication; DEX: Dysexecutive Questionnaire–Self-report. Effect size measured by Cohen’s *d* (0.2 = *small*; 0.5 = *moderate*; 0.8 = *large*).

* *p* < 0.05.

#### Clinically significant change

As shown in Table 6, the clinically significant change scores differed between the NeuroACT and the TAU group. The NeuroACT group showed about twice as many participants recovering from stress (PSS) and about three times more who had a clinically significant improvement compared with the TAU group. Regarding quality of life (SWLS), nearly three times as many participants showed recovery, while a clinically significant improvement was observed with a 4:0 ratio in the NeuroACT group compared with the TAU group. More participants showed no clinically significant improvement in the TAU group compared with the NeuroACT group.

**Table 6.** Clinically significant change in stress (PSS) and quality of life (SWLS) (primary outcomes) against NeuroACT versus TAU from T1 to T3.

Classification	PSS		SWLS	
	NeuroACT (*n* = 16)	TAU (*n* = 18)	NeuroACT (*n* = 16)	TAU (*n* = 18)
	*n* (%)	*n* (%)	*n* (%)	*n* (%)
Recovered	8 (50)	5 (28)	6 (38)	2 (11)
Improved	6 (38)	2 (11)	4 (25)	0 (0)
Unimproved	2 (13)	7 (39)	7 (44)	13 (72)

PSS: Perceived Stress Scale; SWLS: Satisfaction with Life Scale; TAU: treatment as usual; recovered: clinically significant change—below or above cut-off score; improved: clinically significant change—2 *SD*s below or above the group mean; unimproved: Failed to change 2 *SD*s from group mean.

## Discussion

The current randomized controlled pilot study evaluated the feasibility and preliminary effectiveness of an adapted NeuroACT for autistic adults in a psychiatric outpatient setting. Results indicated overall good treatment feasibility, where most participants completed the treatment alongside high treatment credibility ratings and satisfactory measurement fulfillment. Analyses of effects showed statistically significantly improved primary outcomes of perceived stress and quality of life, with moderate to large effect sizes, in the NeuroACT group compared with the TAU group. In the secondary outcomes, reduced psychological inflexibility, cognitive fusion, cognitive and behavioral avoidance, and AM were statistically significant, with moderate to large effect sizes, in the NeuroACT group compared with the TAU group. Moreover, clinically significant changes in perceived stress and quality of life were observed in favor of the NeuroACT group compared with the TAU group. However, no statistically significant differences were observed between the two groups in depressive symptoms, anxiety, sleep problems, the second quality of life measure, functional impairment, social aspects of autism, or executive difficulties. Furthermore, dropout rates were slightly higher in the NeuroACT group compared with the TAU group. Where significant changes were found, the largest effect sizes were noticed in perceived stress, cognitive and behavioral avoidance, and cognitive fusion (Cohen’s *d* = 1.02–1.24). Significant improvements occurred from T1 to T2, except in AM, where changes were observed from T2 to T3, and quality of life, where changes were equally distributed between T1 and T2 compared with T2 and T3. Improvements from T1 to T2 were maintained or further ameliorated to T3. The results suggest that NeuroACT is a promising treatment for autistic adults with co-existing stress and reduced quality of life and that more extensive evaluations are warranted.

To our knowledge, this is the first RCT to indicate that autistic adults with high perceived stress and reduced quality of life could benefit from ACT. The improvements in several outcomes are in line with research on CBT and mindfulness practice for autistic adults, suggesting that psychological treatments could positively influence mental health and improve quality of life (K. B. Beck et al., 2020; Cachia et al., 2016; Weston et al., 2016). ACT for autistic individuals is mainly based on mindfulness training from a functional analytic perspective (i.e. using mindfulness skills to overcome obstacles and pursue personally chosen values and goals). NeuroACT uses treatment techniques and content, such as motivation, acceptance, perspective-taking on thoughts, and psychoeducation to create psychological flexibility (Hayes, 2021). Enhancing psychological flexibility may be especially important in autistic individuals since insufficient emotion regulation skills alongside problematic behavioral avoidance are common problems that affect mental health negatively (Mazefsky, 2015). ACT skills help motivate participants to overcome obstructive thoughts, difficult emotions, and a lack of values clarity. In this study, the reduced obstacles of perceived stress and behavioral avoidance, alongside the improved quality of life, may thus indicate a broadening of the participants’ behavior repertoire, enhancing a sense of meaning in everyday life.

The overall significant interaction effect of the SWLS but not the QOLI, both measuring quality of life, may be associated with the more durable sub-areas of the QOLI, such as economic status, neighborhood well-being, and family-related concerns. The SWLS measures subjective quality of life and overall well-being, potentially reflecting a more general sense of meaning and purpose than the QOLI. Replication studies and prolonged follow-up might further evaluate the eventual effects of using these instruments in autistic adults.

While no statistically significant changes were observed in social aspects of autism, such as social cognition, social awareness, or communication, significant improvements were found in AM, related to cognitive and behavioral inflexibility. The finding is in line with research on modified CBT for autistic individuals, which has observed changes in the SRS subscales of social motivation and AM but not overall social functioning (Bemmer et al., 2021). Also, this result may align with the NeuroACT program’s overarching treatment goal of making social difficulties less of an obstacle to being active in social relationships without training the social skills themselves. For example, better handling obstructive thoughts, such as “I’m odd” or “I can’t have social relations,” indicated by increased cognitive defusion in this study, social situations might be perceived as less stressful. Also, research suggests that some autistic core challenges, such as social motivation, are not independent of emotional distress, indicating that reducing stress and anxiety in autistic individuals may facilitate social interaction (South et al., 2017). Social skills training is crucial in developing specific social behaviors (Choque Olsson et al., 2017). However, this study’s data suggest that training skills to handle obstructive thoughts and fears of social situations may complement social skills training, helping autistic individuals manage some difficulties due to stress and high anxiety levels.

Moreover, in ACT for autistic individuals, the goal is not to “treat autism,” but to alleviate some core challenges that are thought to be exacerbated by mental health problems. ACT aims to change how the individual perceives his or her autistic functioning. By doing this, the individual may be able to act more flexibly in the presence of autism. Consequently, the observed changes in one subscale of the self-rated version of the SRS, as a result of the intervention, may reflect an altered perception but not necessarily an actual change in autistic core difficulties. Future studies may further evaluate these two treatment approaches in autistic individuals; social skills training, developing skills to handle thoughts and emotions, or a combination of both.

Although promising, the current study has limitations. First, a small sample size reduces statistical power and increases the risk of type 2 error. Second, there might be a risk of type 1 error regarding the statistically significant efficacy measures due to the vast number of measures included, increasing the risk of a multiple testing effect. However, several outcome measures’ changes align with previous research, suggesting that the results do not depend just on chance. Third, the outcome measures relied on participant’s self-report, and no objective or independent criteria were used, increasing the risk of over- or underestimating individual progress. Fourth, the study was conducted within a psychiatric outpatient clinic, so the generalizability outside this setting may be limited. Fifth, the study design did not control for group effects. Therefore, changes observed may not be entirely due to ACT but an effect of the intervention’s group setting. Sixth, the participants included in the study all had average or above-average intellectual capacity, so generalization of the results to autistic adults with lower intellectual capacity cannot be made.

## Concluding remarks

Overall, this study results indicate that the NeuroACT program may be a feasible and beneficial treatment for autistic adults with comorbid stress and reduced quality of life. However, for future research, to increase the integrity of the results, large-scale studies with blinded assessments, control of treatment adherence, and group aspects of the intervention, alongside qualitative feedback from participants, are needed to explore the potential benefits of NeuroACT in autistic adults further. In addition, evaluating the effect of potential mediators and moderators of change, such as sex, cognitive profile, psychological inflexibility, behavioral avoidance, or adherence to homework assignments, is warranted.
